# Long-term effects of left-behind experience on adult depression: Social trust as mediating factor

**DOI:** 10.3389/fpubh.2022.957324

**Published:** 2022-09-09

**Authors:** Yan Wang, Shuai Yang, Feng Wang, Zhijun Liu

**Affiliations:** ^1^Department of Sociology, Zhejiang University, Hangzhou, China; ^2^Department of Health Management and Policy, School of Public Health, Hangzhou Normal University, Hangzhou, China

**Keywords:** left-behind experience, long-term effects, depression, social trust, adult

## Abstract

**Background:**

Despite much attention paid to the mental health of left-behind children, there has not been sufficient research on whether and how left-behind experiences have long-term effects on adults among the general population. This paper aims to evaluate the long-term effects of left-behind experience on adult psychological depression.

**Methods:**

By using the China Labor-force Dynamics Survey in 2018 (CLDS 2018), we assessed depression by the Center for Epidemiological Studies, Depression Scale (CES-D) and used a cut-off score of 20 for detecting depression (Yes = 1, No = 0). The Binomial logistic regression was used to compare the odds ratio across groups. We used the KHB method in the mediation analysis, to measure the indirect effect of social trust on the relationship between left-behind experience and depression.

**Results:**

The rate of depression (χ2 = 17.94, *p* < 0.001) for the children who have left-behind experience (LBE) (10.87%) was higher than the children who have non-left-behind experience (N-LBE) (6.37%). The rate of social trust (χ2 = 27.51, *p* < 0.001) of LBE (65.70%) was lower than N-LBE (75.05%). Compared with the other three groups, left-behind experience occurred in preschool (OR = 2.07, *p* < 0.001, 95% CI = [1.45, 2.97]) was more likely to suffer from depression. The indirect effect of social trust (OR = 1.06, *p* < 0.01, 95% CI = [1.02, 1.10]) is significantly on the relationship between LBE and psychological depression, with the total effect (OR = 1.71, *p* < 0.001, 95% CI = [1.27, 2.31]) and direct effect (OR = 1.62, *p* < 0.01, 95% CI = [1.20, 2.18]) are both significantly. The proportion of indirect effect in the total effect is 10.69%.

**Conclusion:**

The left-behind experience that occurred in childhood has a significantly negative effect on adult psychological depression, in which preschool left-behind experience played the most critical role. Social trust is the mediating factor associated with left-behind experience and psychological depression. To mitigate the long-term effects of the left-behind experience on psychological depression, parents need to be prudent about the decision-making of migration in the preschool stage of their children. and subsequent policies should strengthen social work targeting vulnerable youth groups especially those with left-behind experience at an early age in terms of their psychological depression.

## Introduction

Left-behind experience of adults refers to those whose parents or one of their parents used to be migrants, and could not live with parents in their areas of origin during childhood (1). As a rapidly growing international phenomenon, left-behind children are rooted in the context of globalization and urbanization. Transnational labor migration is the primary motivator for the emergence of left-behind children in Southeast Asia (2, 3), Africa (4, 5), the Caribbean (6, 7), and other less developed areas. The main reason for left-behind children in China, however, is domestic trans-regional migration. The scale of left-behind children in China is much larger due to China's large population base and the lower difficulty of trans-regional migration compared to transnational migration. According to the United Nations Children's Fund's 2018 report, there were 28.3 million and 40.5 million left-behind children in China's urban and rural areas, respectively (8). More attention should be paid to this demographic trend. Meanwhile, numerous studies have found that the absence of parents, the stage of being left behind, and the duration of being left behind all have negative effects on individual health, particularly mental health and psychosocial wellbeing (9–12).

Several studies have found that left-behind children are at a disadvantage in terms of mental health when compared to non-left-behind children (13–18), particularly in terms of depression (19–22). A systematic review found a higher incidence of psychological depression in left-behind children ranging from 12.1 to 51.4% when compared to age-matched non-left-behind children (23). Furthermore, Fellmeth et al. collected data from 91 Chinese studies and discovered that left-behind children had a higher risk of depression and depression scores than non-left-behind children (11). Simultaneously, a few studies have reported different results, implying that there is no significant difference in mental health among child groups with different left-behind statuses (18, 24).

Current studies mainly focus on the short-term effect of left-behind experience on adolescents, ignoring the long-term effects of left-behind experience on adults. Some research has indicated that the left-behind experience has long-term effects, but in most cases, long-term effects mean lasting negative effects on adolescents, rather than on adults (25–27). According to one study, even after the status of left-behind has ended, the influence of the left-behind experience is still evident in adult mental health (28).

Some studies have focused on the long-term effects of childhood left-behind experiences on college students. According to these research, college students who had left-behind experience in childhood underperform other college students in terms of happiness (29–31), psychological resilience (32–34), depression (34–36), social anxiety (37, 38) and other aspects. These studies give critical evidence regarding the long-term effects of being left behind.

The college student samples, however, have clear weaknesses in examining the long-term effects of the left-behind experience. On the one hand, the college experience can help to mitigate the negative effects of left-behind experience. According to one study, a greater level of education, particularly college experience, has a considerable protective effect on mental health (39). On the other hand, left-behind children who have higher education outcomes outperformed other left-behind children in terms of family socioeconomic status and childhood academic performance (40). Therefore, to examine the long-term effects of left-behind experience, we need to start with the general population rather than college students because of the internal heterogeneity of left-behind children.

A growing number of studies have reported that micro-mechanisms about the key mediating factors between left-behind experiences and mental health. Relevant studies claimed that left-behind children have lower scores, in psychological resilience (41–43), self-concept clarity (44), parent-child communication (45–47), compared with non-left-behind children, resulting in a higher risk of psychological problems. These studies, however, exclude the influence of meso-level variables, which should to be viewed as the protective factor linked with depression (48). A few studies have found that social capital is a protective factor in children's mental health, and showed that left-behind children have lower social capital, led to higher degrees of psychological problems (49, 50).

Social capital is defined by social networks, norms of reciprocity, and trust (51). Scholars generally operationalize social capital into family capital (49, 50, 52), school capital (50, 52), community capital (49, 50), and find that left-behind children underperform non-left-behind children in the parent-child relationship and teacher-student relationship, leading negative impact on their mental health.

However, there are two deficiencies in the above research's operationalization and measurement. First, these studies only focus on the objective dimension such as social network, while ignoring the social trust as the subjective dimension of social capital. Secondly, scholars now use variables from the levels of family and school to quantify social capital, and there is a shortage of social-level factors. To address the aforementioned two shortcomings, this article seeks to evaluate social trust in the general population in order to investigate the relation of the left-behind experience affecting psychological depression.

In short, there is a large amount of research on the depression of left-behind children, however, a small quantity of literature has focused on the long-term effects of left-behind experience on adults. Some studies have focused on the long-term effects of childhood left-behind experiences on college students. But using college student samples will ignore the internal heterogeneity of left-behind children. In addition, very few studies have explored social trust as mediating factor in the relationship between left-behind experience and depression. This study aims to fill these gaps above by examining the effects of left-behind experiences on depression in adults among the general population with social trust as mediating factor.

Based on the review of current literature, we listed the following research hypotheses:

**H1:** LBE has a higher probability of depression than N-LBE.

**H2:** LBE has a lower probability of social trust than N-LBE.

**H3:** LBE affects psychological depression through social trust. Adults who have left-behind experience have lower levels of social trust, leading to an increased risk of psychological depression.

## Materials and methods

### Participants and procedure

This study used data from the China Labor-force Dynamics Survey 2018 (CLDS 2018). The data, collected by the Social Science Survey Center at Sun Yat-sen University, covers a wide range of research topics, including education, work, migration, health, social participation, economic activity, and grassroots organizations. The sample covers 29 provinces and cities across China (except Hong Kong Special Administrative Region, Macao Special Administrative Region, Taiwan, Tibet Autonomous Region, and Hainan province). In terms of the Sampling method, PPS (Probability Proportion to Size Sampling) method was adopted to complete the investigation of 16,537 labor individuals (53, 54). Since China gradually lifted restrictions on population movement after the 1980s, which lead to the emergence of labor migration and left-behind children, this paper mainly focuses on the participants born after the 1980s.

### Depression

Depression was assessed by the Center for Epidemiological Studies, Depression Scale (CES-D) (55). This scale consists of 20 items, and each item is scored on a 4-point Likert scale: 0 (<1 day), 1 (1–2 days), 2 (3–4 days), 3 (5–7 days), which aims to evaluate how often people have felt in that way, such as “*people were unfriendly*,” during the past week. So that the possible range of scores on the CES-D scale is 0 to 60, with the higher scores indicating a greater risk of depression (55–57). For the original CES-D scale, the author suggested a total score equal to or above 16 indicates the participant is at risk for clinical depression (55). However, a lot of studies have evaluated the diagnostic accuracy of the CES-D scale for detecting depression in the general population and have proposed various cutoff scores, such as 18,21,22 (58–60). After using a meta-analytic approach, the present study used a cut-off score of 20 for detecting depression (61). According to this, we generated a dummy variable about depression (Yes = 1, No = 0). This measure of the CES-D scale has been used across age group, community, and country samples (57), and validated in a variety of Chinese samples (62–64). The internal consistency (Cronbach's α) for the CES-D scale in this study was 0.949.

### Social trust

Social trust was evaluated by asking the respondents the following question: “In general, do you agree with the statement that most people can be trusted” The answer options were scored on a 4-point Likert scale: 1 (very disagree), 2 (disagree), 3 (agree), 4 (very agree). According to a previous study, we further transform the 4-point Likert scale to a binary variable: 0 (disagree), 1 (agree) (65), and renamed these two groups to 0 (No), 1 (Yes).

### Left-behind experience

Left-behind experience was identified and differentiated by asking the respondents the following questions: “In the stage of preschool, did your father (and/or mother) migrate from original area to another place for work?” (The answer options were “yes,” “no”); “In the stage of primary school, did your father (and/or mother) migrate from original area to another place for work?” (The answer options were “yes,” “no”); “In the stage of middle school, did your father (and/or mother) migrate from original area to another place for work?” (The answer options were “yes,” “no”).These questions differentiated the participants into five categories: (1) N-LBE: in any stage, their father (and/or mother)were not absent; (2) LBE: in any one or more stages, their father (and/or mother)were absent;(3) LBE-PRE: in the stage of preschool, their father (and/or mother)were absent for the first time; (4) LBE-PRI: in the stage of primary school, their father (and/or mother)were absent for the first time; (5) LBE-MID: in the stage of middle school, their father (and/or mother)were absent for the first time. According to the five categories, we generated two variables. One is a dummy variable, “*Left-behind experience status*,” to differentiate N-LBE and LBE, the reference group is N-LBE. Another one is a multiple category variable, the “*Left-behind experience stage*,” to differentiate N-LBE and LBE-PRE, LBE-PRI, and LBE-MID, the reference group is N-LBE. Additionally, we also evaluated the “*Duration of left-behind experience*” for participants by summing their left-behind years in different stages.

### Covariates

Covariates in this study included individual age, gender, education level, marital status, *hukou* status (household registration system) (66), work status (work status refers to whether the participant has ever had a job, and was characterized as “worked” and “Not worked”), parental education level (parental education level measured separately by the highest educational level of the father and mother, and were both characterized as “Illiteracy,” “Primary school,” “Middle school” and “Above Middle”) (9) and family structure (the family structure was measured by whether participants had siblings, and was characterized as “Yes,” “No”) (9). Covariates above have been proved to be correlated with left-behind status and mental health in previous studies.

### Statistical analysis

The Chi-square test and *t*-test were used in descriptive statistics to compare the differences in characteristics across groups. The binomial logistic regression was used for predicting the depression. Models' key independent variables included LBE status, LBE stage, duration of left behind respectively. The KHB-method (67–70) was used to measure the indirect effect of social trust on the relationship between left-behind experience and depression. Data management and all statistical analyses were carried out using Stata16 (Stata Corp LLC., Texas, USA) for windows. *P* < 0.05 was regarded as statistically significant.

## Results

Since this study focused on the depression of adults who had been left behind as children, we eliminated individuals who had not yet graduated from junior middle school at the time of the survey (CLDS 2018), and only participants aged 18 and up were included.

As can be seen in Table 1, the final sample for analysis included 4,056 adults, 3,298 with no left-behind experience were in the N-LBE group, while 758 with left-behind experience were in the LBE group. Table 1 also showed the duration of left-behind (9.56 ± 5.07) of LBE. In addition, the mean age of N-LBE was 29.6 (SD = 5.83) being older than LBE (mean = 27.3, SD = 6). Compared with N-LBE, more participants in LBE were single (43.27%) and not worked (25.99%). Overall, there were significant differences in terms of father's education (*t* = 8.06, *p* < 0.05) while there were no significant differences in terms of education, mother's education, gender, *hukou* status, and the sibling number among participants in N-LBE and LBE.

**Table 1:** Sample characteristics by left-behind experience status, *N* (%)/Mean (SD).

	**N-LBE** **(*N* = 3,298)**	**LBE** **(*N* = 758)**	***t*** **or χ2**	***P*** **-Value**
**Education**			9.67	0.085
Illiteracy	72 (2.19)	19 (2.51)		
Primary school	315 (9.58)	62 (8.19)		
Middle school	1,056 (32.13)	220 (29.06)		
High school	726 (22.09)	203 (26.82)		
College degree	450 (13.69)	106 (14.00)		
Bachelor and above	668 (20.32)	147 (19.42)		
**Education of father**			8.06	0.045
Illiteracy	338 (10.73)	57 (8.03)		
Primary school	1,094 (34.72)	242 (34.08)		
Middle school	1,017 (32.28)	261 (36.76)		
Middle school and above	702 (22.28)	150 (21.13)		
**Education of mother**			6.71	0.082
Illiteracy	758 (24.08)	154 (21.54)		
Primary school	1,189 (37.77)	306 (42.80)		
Middle school	767 (24.36)	168 (23.50)		
Middle school and above	434 (13.79)	87 (12.17)		
**Gender**			2.15	0.142
Male	1,491 (45.21)	365 (48.15)		
Female	1,807 (54.79)	393 (51.85)		
**Marital status**			24.84	<0.001
Married	2,187 (66.33)	430 (56.73)		
Single	1,110 (33.67)	328 (43.27)		
***Hukou*** **status**			2.18	0.140
Urban	661 (20.09)	134 (17.72)		
Rural	2,629 (79.91)	622 (82.28)		
**Work status**			10.62	0.001
Worked	2,619 (79.41)	561 (74.01)		
Not worked	679 (20.59)	197 (25.99)		
**Any siblings**			0.84	0.360
Yes	2,534 (76.95)	595 (78.50)		
No	759 (23.05)	163 (21.50)		
Duration of center-behind		9.56 (5.07)		
Age	29.6 (5.83)	27.3 (6.00)	9.97	<0.001

Using the chi-square test for categorical variables and the t-test for continuous variables.

N-LBE, in any stage, their father (and/or mother) were not absent; LBE, in any one or more stages, their father (and/or mother) were absent; LBE-PRE, in the stage of preschool, their father (and/or mother) were absent for the first time; LBE-PRI, in the stage of primary school, their father (and/or mother) were absent for the first time; LBE-MID, in the stage of middle school, their father (and/or mother) were absent for the first time.

Table 2 shows, there were significant differences in psychological depression and social trust among the two adult groups. The rate of depression (χ2 = 17.94, p < 0.001) of LBE (10.87%) was higher than N-LBE (6.37%). The rate of social trust (χ2 = 27.51, p < 0.001) of LBE (65.70%) was lower than N-LBE (75.05%). H1 and H2 were proved.

**Table 2:** Depression and social trust by left-behind experience status, *N* (%).

	**N-LBE** **(*N* = 3,298)**	**LBE** **(*N* = 758)**	**χ2**	***P*** **-Value**
**Depression**			17.94	<0.001
Yes	199 (6.37)	80 (10.87)		
No	2,923 (93.63)	656 (89.13)		
**Social trust**			27.51	<0.001
Yes	2,475 (75.05)	498 (65.70)		
No	823 (24.95)	260 (34.30)		

N-LBE, in any stage, their father (and/or mother) were not absent; LBE, in any one or more stages, their father (and/or mother) were absent.

Table 3 presents, for the LBE group, the first time they experienced parental absence, 434 (57.26%) occurred in preschool, 117 (15.44%) occurred in primary school, and 207 (27.31%) occurred in middle school. In addition, there were significant differences in psychological depression and social trust among the four groups. The rate of depression (χ2 = 23.26, p< 0.001) of LBE-PRE was the highest (12.83%) compared with LBE-PRE (8.55%), LBE-MID (8.25%), and N-LBE (6.37%). In contrast, the rate of social trust (χ2 = 29.20, p< 0.001) of N-LBE was the highest (75.05%) compared with LBE-PRE (67.28%), LBE-MID (61.54%) and N-LBE (64.73%).

**Table 3:** Depression and social trust by left-behind experience stage, *N* (%).

	**N-LBE** **(*N* = 3,298)**	**LBE (*****N*** = **758)**			**χ2**	***P*** **-Value**
		**LBE-PRE (*N* = 434)**	**LBE-PRI (*N* = 117)**	**LBE-MID (*N* = 207)**		
**Depression**					23.36	<0.001
Yes	199 (6.37)	53 (12.83)	10 (8.55)	17 (8.25)		
No	2,923 (93.63)	360 (87.17)	107 (91.45)	189 (91.75)		
**Social trust**					29.20	<0.001
Yes	2,475 (75.05)	292 (67.28)	72 (61.54)	134 (64.73)		
No	823 (24.95)	142 (32.72)	45 (38.46)	73 (35.27)		

N-LBE, in any stage, their father (and/or mother) were not absent; LBE, in any one or more stages, their father (and/or mother) were absent; LBE-PRE, in the stage of preschool, their father (and/or mother) were absent for the first time; LBE-PRI, in the stage of primary school, their father (and/or mother)were absent for the first time; LBE-MID, in the stage of middle school, their father (and/or mother)were absent for the first time.

Table 4 shows, with controlling participants' demographic characteristics in M1, the left-behind experience that occurred in childhood has a significant effect on their psychological depression, LBE (OR = 1.62, p < 0.01, 95% CI = [1.20, 2.18]) was more likely to suffer depression. M2 shows, left-behind experiences, which firstly occurred in preschool, significantly predicted depression. Compared with the other three groups, LBE-PRE (OR = 2.07, p < 0.001, 95% CI = [1.45, 2.97]) was more likely to suffer from depression. M3 shows, that the variable of the duration of left behind had no significant effect on the psychological depression among the LBE group.

**Table 4:** Logistic analysis for depression, OR (95% CI).

	**M1**	**M2**	**M3**
**LBE status (*****Ref*** **= N-LBE)**			
LBE	1.62 (1.20, 2.18)^**^		
**LBE stage (*****Ref*** **= N-LBE)**			
LBE-PRE		2.07 (1.45, 2.97)^***^	
LBE-PRI		0.99 (0.47, 2.10)	
LBE-MID		1.25 (0.74, 2.13)	
Duration of left-behind			1.02 (0.97, 1.08)
**Social trust (*****Ref*** **= No)**			
Yes	0.51 (0.39, 0.67)^***^	0.51 (0.39, 0.66)^***^	0.43 (0.25, 0.73)^**^
Age	1.01 (0.98, 1.04)	1.01 (0.98, 1.04)	1.02 (0.96, 1.09)
**Gender (*****Ref*** **= female)**			
Male	0.89 (0.68, 1.17)	0.89 (0.68, 1.17)	0.83 (0.47, 1.46)
**Education (*****Ref*** **= illiteracy)**			
Primary school	0.45 (0.24, 0.87)^*^	0.46 (0.24, 0.88)^*^	0.24 (0.06, 0.96)^*^
Middle school	0.25 (0.14, 0.48)^***^	0.26 (0.14, 0.48)^***^	0.24 (0.07, 0.89)^*^
High school	0.25 (0.13, 0.49)^***^	0.25 (0.13, 0.49)^***^	0.22 (0.06, 0.85)^*^
College degree	0.21 (0.10, 0.44)^***^	0.21 (0.10, 0.44)^***^	0.17 (0.04, 0.73)^*^
Bachelor and above	0.16 (0.08, 0.34)^***^	0.16 (0.08, 0.34)^***^	0.16 (0.04, 0.75)^*^
**Marital status (*****Ref*** **= single)**			
Married	0.67 (0.47, 0.95)^*^	0.66 (0.47, 0.94)^*^	0.93 (0.43, 2.01)
***Hukou*** **status (*****Ref*** **= rural)**			
Urban	1.37 (0.92, 2.03)	1.37 (0.92, 2.04)	0.65 (0.25, 1.66)
**Education of father (*****Ref*** **= illiteracy)**			
Primary school	0.79 (0.50, 1.26)	0.79 (0.50, 1.26)	0.97 (0.37, 2.57)
Middle school	0.75 (0.46, 1.23)	0.75 (0.46, 1.23)	0.85 (0.31, 2.33)
High school and above	0.57 (0.32, 1.02)	0.58 (0.32, 1.03)	0.57 (0.17, 1.92)
**Education of mother (*****Ref*** **= illiteracy)**			
Primary school	1.07 (0.73, 1.55)	1.04 (0.71, 1.52)	0.95 (0.46, 1.96)
Middle school	1.12 (0.72, 1.75)	1.09 (0.70, 1.70)	1.01 (0.42, 2.40)
High school and above	1.10 (0.61, 2.00)	1.08 (0.59, 1.96)	0.89 (0.27, 2.99)
**Work status (*****Ref*** **= not worked)**			
Worked	1.12 (0.79, 1.59)	1.15 (0.81, 1.64)	1.45 (0.67, 3.14)
**Any siblings (*****Ref*** **= no)**			
Yes	0.82 (0.56, 1.20)	0.80 (0.55, 1.18)	1.00 (0.45, 2.25)

* p < 0.05,

** p < 0.01,

*** p < 0.001;

OR, odds ratio; CI, confidence intervals.

N-LBE, in any stage, their father (and/or mother) were not absent; LBE, in any one or more stages, their father (and/or mother) were absent; LBE-PRE, in the stage of preschool, their father (and/or mother) were absent for the first time; LBE-PRI, in the stage of primary school, their father (and/or mother) were absent for the first time; LBE-MID, in the stage of middle school, their father (and/or mother) were absent for the first time.

M1, The dependent variable is depression, and the key independent variable is LBE status; AIC (1,828.06), BIC (1,951.90), N (3,612), Pseudo R2(0.046); M2, The dependent variable is depression, and the key independent variable is LBE stage; AIC (1,826.93), BIC (1,963.16), N (3,612), Pseudo R2(0.048); M3, The dependent variable is depression, and the key independent variable is the duration of left behind among the LBE group; AIC (460.11), BIC (550.29), N (671), Pseudo R2(0.064).

Table 5 presents the results of KHB method, with participants demographic characteristics in control, the indirect effect of social trust (OR = 1.06, *p* < 0.01, 95% CI = [1.02, 1.10]) is significantly on the relationship between LBE and psychological depression, with the total effect (OR = 1.71, *p* < 0.001, 95% CI = [1.27, 2.31]) and direct effect (OR = 1.62, *p* < 0.01, 95% CI = [1.20, 2.18]) are both significantly. The con-founding percentage, which means the proportion of indirect effect in the total effect, is 10.69%. H3 was proved.

**Table 5:** Examining the indirect effect of social trust by using the KHB method.

	**OR**	**SE**	***P***	**95%CI**	**Con-founding percentage**
**LBE (*****Ref*** **= N-LBE)**					10.69 %
Total effect	1.71	0.26	<0.001	(1.27, 2.31)	
Direct effect	1.62	0.25	0.002	(1.20, 2.18)	
Indirect effect	1.06	0.02	0.001	(1.02, 1.10)	
**LBE-PRE (*****Ref*** **= N-LBE)**					7.06 %
Total effect	2.19	0.40	<0.001	(1.53, 3.14)	
Direct effect	2.07	0.38	<0.001	(1.45, 2.97)	
Indirect effect	1.06	0.04	0.182	(0.98, 1.15)	
**LBE-PRI (*****Ref*** **= N-LBE)**					110.99 %
Total effect	1.08	0.41	0.844	(0.51, 2.28)	
Direct effect	0.99	0.38	0.983	(0.47, 2.10)	
Indirect effect	1.09	0.05	0.053	(1.00, 1.18)	
**LBE-MID (*****Ref*** **= N-LBE)**					17.80 %
Total effect	1.32	0.36	0.310	(0.78, 2.23)	
Direct effect	1.25	0.34	0.404	(0.74, 2.13)	
Indirect effect	1.05	0.04	0.235	(0.97, 1.14)	
**Duration of left-behind**					5.72 %
Total effect	1.03	0.03	0.341	(0.97, 1.08)	
Direct effect	1.02	0.03	0.369	(0.97, 1.08)	
Indirect effect	1.00	0.00	0.649	(1.00, 1.01)	

OR, odds ratio; SE, standard error; p, significance level; CI, confidence intervals; Con-founding percentage, proportion of indirect effect in the total effect.

N-LBE, in any stage, their father (and/or mother) were not absent; LBE, in any one or more stages, their father (and/or mother) were absent; LBE-PRE, in the stage of preschool, their father (and/or mother) were absent for the first time; LBE-PRI, in the stage of primary school, their father (and/or mother) were absent for the first time; LBE-MID, in the stage of middle school, their father (and/or mother) were absent for the first time.

In addition, as Table 5 showed, social trust has no significant mediating effect on the relationship between the left-behind stage (LBE-PRE, LBE-PRI, and LBE-MID) and depression. And there was no significant mediating effect between the duration of left-behind and depression.

## Discussion

This study aims to examine the effect of left-behind experience, including the left-behind stage and duration of left-behind, on psychological depression of N-LBE and LBE among the general population, and to explain the mechanism using social trust as a mediating factor.

Firstly, we discovered participants in the LBE group have a larger proportion of psychological depression than N-LBE. Several prior Chinese studies have validated this finding (19, 20, 23, 44, 71–73). In addition, the lower the level of social trust, the higher the risk of depression while LBE was lower than N-LBE in social trust. Previous research also has revealed that the intimate relationship pattern with others in the early stages influences the social relationship pattern in subsequent growth (74–76). According to the KHB method, social trust plays a mediating role between left-behind experience and psychological depression. It is worth noting that adults who have been left behind have lower levels of social trust, leading to an increased risk of psychological depression. Many prior studies indicated that factors from the social level need to be considered in future research (43, 49, 50, 77–81).

Secondly, regression analysis results suggest that LBE significantly increased the probability of depression, with preschool left-behind (LBE-PRE) playing the most important impact. However, the duration of left behind did not significantly predict psychological depression. These findings showed the “earlier or later” effect, which refers to the stage influence of left-behind experience (28), was proved in our study, and demonstrated the negative effect of left-behind experience on adult psychological depression mainly comes from the preschool stage. It has been proposed that parental absence in early childhood has more long-term detrimental impacts on the individual (82). According to attachment theory, children's early experiences are strongly linked to the development of subsequent psychological problems (74). As a result, we believe that parental absence at an early age will result in developmental delays and long-term impacts on mental health in children (83–87).

Thirdly, contrary to the previous study (28), the present study shows that the “cumulative or temporary” effect, which refers to the length of left-behind time, was not significant in predicting the psychological depression of adults. The reason is that the effect of duration on depression is an internal comparison within the LBE group without using the N-LBE as the reference group, and the variations in depression among LBE are quite minor.

Fourthly, the results above suggest that the “earlier or later” effect of left-behind experiences is more important than “cumulative or temporary” effect in predicting adult depression. Many Chinese parents believe that they can entrust their children to grandparents for care during the preschool stage when is easier to after-school tutoring and carries less of the educational burden than other stages. They also believe that short-term separation from their children is less harmful than long-term absence. Our findings in this research will be useful for Chinese parents making migration decisions.

Lastly, this present study showed the long-term effects of left-behind experiences on depression in adults among the general population. This shows that researchers should pay attention not just to children who are left behind, but also to adults who have left-behind experiences. In recent years, the Chinese government has paid greater attention to the youth group. In 2017, the State Council of China enacted the “Medium and Long-term Youth Development Plan (2016-2025),” which included youth health as one of 10 priority sectors of youth development (88). As a result, we believe that future policies should place a greater emphasis on vulnerable youth groups, particularly those who have been left behind at a young age.

There are some limitations to this study. First, we did not further distinguish the custodial type, such as single-parent custody (father absence or mother absence) and grandparent custody (father absence and mother absence). However, the custodial type may play a moderating role in the impact of left-behind experience on psychological depression. Considering that parental migration status and custodial types are both crucial perspectives on left-behind children study, we will examine this separately in future research. Second, as previously stated, some research revealed that social trust was secondary to social capital, which acted as a mediator between left-behind experience and children's development (40, 49, 50, 52). In our next study, we will attempt to use the social capital as mechanism to analyze the long-term effects of left-behind experience on individual health. Third, there are several confounding factors impact the strength of the long-term effect of left-behind experience on adult depression, and the reliability of self-report data will also be altered as the time lengthens. Although our sample is drawn from a nationwide sample survey, as cross-sectional data, it has limitations in showing causation when compared to panel data.

## Conclusion

The present study focuses on the long-term effects of left-behind experience on adults among the general population. Childhood left-behind experience has a considerable detrimental effect on adult psychological depression, with preschool left-behind experience playing the most crucial role. Social trust is the mediating factor between left-behind experience and psychological depression. Adults who have been left behind have lower levels of social trust, which increases their risk of psychological depression. To mitigate the long-term effects of the left-behind experience on psychological depression, parents must be cautious when making migration decisions for their preschool-aged children. Simultaneously, we propose that the government and future policies pay greater attention to the youth group with childhood left-behind experience, as well as strengthen social work targeting the vulnerable youth group in terms of psychological depression.

## Data availability statement

The original contributions presented in the study are included in the article/supplementary material, further inquiries can be directed to the corresponding author.

## Ethics statement

The studies involving human participants were reviewed and approved by Ethics Committee at Department of Sociology, Sun Yat-sen University. The patients/participants provided their written informed consent to participate in this study.

## Author contributions

ZL conceived the design of the study. YW completed the first draft of this article. SY participated in data analysis. FW revised the manuscript and made valuable suggestions on scholarly writing. All authors have read and agreed to the published version of the manuscript.

## Funding

This research was supported by Zhijiang Youth Project of Zhejiang Province (18ZJQN01YB) and National Natural Science Foundation of China (71774138).

## Conflict of interest

The authors declare that the research was conducted in the absence of any commercial or financial relationships that could be construed as a potential conflict of interest.

## Publisher's note

All claims expressed in this article are solely those of the authors and do not necessarily represent those of their affiliated organizations, or those of the publisher, the editors and the reviewers. Any product that may be evaluated in this article, or claim that may be made by its manufacturer, is not guaranteed or endorsed by the publisher.

## References

[1] Lv L Yan F Duan C Cheng M. Changing patterns and development challenges of child population in China. Populat Res. (2018) 42:65–78. 32471396

[2] Lam T Yeoh BSA. Parental migration and disruptions in everyday life: reactions of left-behind children in Southeast Asia. J Ethn Migr Stud. (2019) 45:3085–104. 10.1080/1369183X.2018.1547022 31827371 PMC6874285

[3] Graham E Jordan LP. Migrant parents and the psychological wellbeing of left-behind children in Southeast Asia. J Marr Fam. (2011) 73:763–87. 10.1111/j.1741-3737.2011.00844.x 22163371 PMC3229683

[4] GonzÁlez-ferrer A BaizÁn P Beauchemin C Adserà A Tienda M. Child-parent separations among Senegalese migrants to Europe: migration strategies or cultural arrangements? Annal Am Acad Politic Soc Sci. (2012) 643:106–133. 10.1177/0002716212444846

[5] Chuong C Operario D. Challenging household dynamics: Impact of orphanhood, parental absence, and children's living arrangements on education in South Africa. Glob Public Health. (2012) 7:42–57. 10.1080/17441692.2011.574147 21644120

[6] Pottinger AM Stair AG Brown SW. A counselling framework for caribbean children and families who have experienced migratory separation and reunion. Int J Adv Counsell. (2008) 30:15–24. 10.1007/s10447-007-9041-x

[7] Dillon M Walsh CA. Left behind: the experiences of children of the caribbean whose parents have migrated. J Comp Fam Stud. (2012) 43:871–902. 10.3138/jcfs.43.6.871

[8] UNICEF. (2018). Available online at: https://www.unicef.cn/en/atlas-2018-en. (accessed May 01, 2022).

[9] Wang F Lin L Lu J Cai J Xu J Zhou X. Mental health and substance use in urban left-behind children in China: a growing problem. Child Youth Serv Rev. (2020) 116:105135. 10.1016/j.childyouth.2020.105135

[10] Zhao C Wang F Zhou X Jiang M Hesketh T. Impact of parental migration on psychosocial wellbeing of children left behind: a qualitative study in rural China. Int J Equity Health. (2018) 17:95. 10.1186/s12939-018-0795-z 29903019 PMC6003177

[11] Fellmeth G Rose-Clarke K Zhao C Busert LK Zheng Y Massazza A. Health impacts of parental migration on left-behind children and adolescents: a systematic review and meta-analysis. Lancet. (2018) 392:2567–82. 10.1016/S0140-6736(18)32558-3 30528471 PMC6294734

[12] Zhao F Yu G. Parental migration and rural left-behind children's mental health in China: a meta-analysis based on mental health test. J Child Fam Stud. (2016) 25:3462–72. 10.1007/s10826-016-0517-3

[13] Tang W Wang G Hu T Dai Q Xu J Yang Y. Mental health and psychosocial problems among Chinese left-behind children: a cross-sectional comparative study. J Affect Disord. (2018) 241:133–41. 10.1016/j.jad.2018.08.017 30121025

[14] Allen B Cisneros EM Tellez A. The children left behind: the impact of parental deportation on mental health. J Child Fam Stud. (2015) 24:386–92. 10.1007/s10826-013-9848-5

[15] Liao C Wu J Zhang J. A factor analysis of the mental health of children left behind: perspective of sense of security. J East China Norm Univ. (2015) 33:88–97. 10.16382/j.cnki.1000-5560.2015.03.012

[16] Adhikari R Jampaklay A Chamratrithirong A Richter K Pattaravanich U Vapattanawong P. The impact of parental migration on the mental health of children left behind. J Immigr Minor Health. (2014) 16:781–9. 10.1007/s10903-013-9809-5 23546615

[17] Graham E Jordan LP Yeoh BSA. Parental migration and the mental health of those who stay behind to care for children in South-East Asia. Soc Sci Med. (2015) 132:225–35. 10.1016/j.socscimed.2014.10.060 25464878 PMC4405005

[18] Tao X Guan H Zhao Y Fan Z. Mental health among left-behind preschool-aged children: preliminary survey of its status and associated risk factors in Rural China. J Int Med Res. (2014). 10.1177/0300060513503922 24345824

[19] Liang Y Wang L Rui G. Depression among left-behind children in China. J Health Psychol. (2017) 22:1897–905. 10.1177/1359105316676333 28810363

[20] He B Fan J Liu N Li H Wang Y Williams J. Depression risk of 'left-behind children' in rural China. Psychiatry Res. (2012) 200:306–12. 10.1016/j.psychres.2012.04.001 22572158

[21] Shen M Gao J Liang Z Wang Y Du Y Stallones L. Parental migration patterns and risk of depression and anxiety disorder among rural children aged 10–18 years in China: a cross-sectional study. BMJ Open. (2015) 5:e7802. 10.1136/bmjopen-2015-007802 26715475 PMC4710829

[22] Zhou M Sun X Huang L Zhang G Kenny K Xue H. Parental migration and left-behind children's depressive symptoms: estimation based on a nationally-representative panel dataset. Int J Environ Res Public Health. (2018) 15:69. 10.3390/ijerph15061069 29795049 PMC6025488

[23] Cheng J Sun YH. Depression and anxiety among left-behind children in China: a systematic review. Child Care Health Dev. (2015) 41:515–23. 10.1111/cch.12221 25495395

[24] Zhou H. The heterogeneous effect of migration on the psychological status of the relative children. Populat Econ. (2016) 16:45–52.

[25] Menjívar C. Long-term Family Separations and Unaccompanied children's lives: a response to Aryah somers: undocumented children in the US. Int Migrat. (2010) 10:234.

[26] Zhao C Wang F Li L Zhou X Hesketh T. Long-term impacts of parental migration on Chinese children's psychosocial wellbeing: mitigating and exacerbating factors. Soc Psychiatry Psychiatr Epidemiol. (2017) 52:669–77. 10.1007/s00127-017-1386-9 28439622 PMC5487538

[27] Lan X Wang W. To be Shy or avoidant? Exploring the longitudinal association between attachment and depressive symptoms among left-behind adolescents in rural China. Personal Individ Differen. (2020) 155:109634. 10.1016/j.paid.2019.109634

[28] Yao Y Zhang S. The last “soul imprint”: how the left-behind duration influence the youth's early subjective wellbeing. Youth Stud. (2018) 18:23–33.

[29] Xu L Fang Q Chen J Wang P Chen J. The relationship between general wellbeing, sense of security and social support in medical college students of left-behind experience. Med Soc. (2012) 25:87–9.

[30] Zhao G Zhang L Zeng L Shan J Ding W. The relationship between child left-home experience, coping style and wellbeing of college students. Chinese J Dis Control Prevent. (2013) 17:636–8.

[31] Zhou H Huang H Liu C Wu H. Impact of left-behind experience in childhood on undergraduates subject wellbeing: the moderating role of parent emotional warmth. Chinese J Clinic Psychol. (2014) 22:893–6. 10.16128/j.cnki.1005-3611.2014.05.077

[32] Pan G Li B Wang J Zhang G Mo Y Tao X. Status of childhood abuse and mental health in college students with left-behind experience. Chinese J Dis Control & Prevent. (2019) 23:840–4. 10.16462/j.cnki.zhjbkz.2019.07.019

[33] Yang Q Cai T Lin J. The effect of left-behind experience on college students' psychological resilience. China J Health Psychol. (2014) 22:272–4. 10.13342/j.cnki.cjhp.2014.02.04729145763

[34] Zhang W Wang Q Zhang Y Zhai J. The influence of left-behind experience on mental resilience and negative emotions in college students. J Psychiatr. (2019) 32:45–8.

[35] He H Zeng Q Wang H. Analysis on left-behind experience influence on depression status among college students with rural Hukou in Beijing. Chinese J Health Educ. (2018) 34:973–8. 10.16168/j.cnki.issn.1002-9982.2018.11.003

[36] Yi S Zhang S. The relationship between childhood abuse and anxiety/depression of university students with left-behind experience: mediating effect of cognitive emotion regulation. Chinese J Health Educ. (2018) 34:920–3. 10.16168/j.cnki.issn.1002-9982.2018.10.014

[37] Yang L Wu D. The relationship between college students' left-home-experience, security and social anxiety. Educat Psychol. (2015) 13:165–6.35350126

[38] Liang J Zhang S Wu Z. Relationship among social anxiety,emotional maltreatment and resilience in rural college students with left-behind experience. Chinese Mental Health J. (2019) 33:64–9.

[39] Xin Z Zhang M He L. Changes in college students' mental health: a cross-temporal meta-analysis. Acta Psychologica Sinica. (2012) 44:664–79. 10.3724/SP.J.1041.2012.00664

[40] Yan J Xu X. Social capital,resilience and education outcomes of left-behind children. J China Agricult Univ (Social Sciences). (2020) 37:96–105. 10.13240/j.cnki.caujsse.2020.02.010

[41] Zhou C Lv Q Yang N Wang F. Left-behind children, parent-child communication and psychological resilience: a structural equation modeling analysis. Int J Environ Res Public Health. (2021) 18:5123. 10.3390/ijerph18105123 34066012 PMC8150701

[42] Wu Y Zhao X Ding X Yang H Qian Z Feng F. prospective study of psychological resilience and depression among left-behind children in China. J Health Psychol. (2015) 22:627–36. 10.1177/1359105315610811 26490625

[43] Fan X Lu M. Testing the effect of perceived social support on left-behind children's mental wellbeing in mainland China: the mediation role of resilience. Child Youth Serv Rev. (2020) 109:104695. 10.1016/j.childyouth.2019.104695

[44] Li Q Wang J Jin T Zhao S. Relationship between self-disclosure and depression in left-behind junior school children: the mediating effect of self-concept clarity and coping styles. Chinese J Clinic Psychol. (2018) 26:747–51. 10.16128/j.cnki.1005-3611.2018.04.025

[45] Wang F Lin L Xu M Li L Lu J Zhou X. Mental health among left-behind children in rural China in relation to parent-child communication. Int J Environ Res Public Health. (2019) 16:1855. 10.3390/ijerph16101855 31130670 PMC6572381

[46] Lu J Lin L Roy B Riley C Wang E Wang K. The impacts of parent-child communication on left-behind children's mental health and suicidal ideation: a cross sectional study in Anhui. Child Youth Serv Rev. (2020) 110:104785. 10.1016/j.childyouth.2020.104785 32801409 PMC7425803

[47] Su S Li X Lin D Xu X Zhu M. Psychological adjustment among left-behind children in rural China: the role of parental migration and parent-child communication. Child Care Health Dev. (2013) 39:162–70. 10.1111/j.1365-2214.2012.01400.x 22708901

[48] Hietanen H Aartsen M Kiuru N Lyyra T Read S. Social engagement from childhood to middle age and the effect of childhood socio-economic status on middle age social engagement: results from the National Child Development study. Ageing Soc. (2016) 36:482–507. 10.1017/S0144686X1400124X

[49] Wu Q Lu D Kang M. Social capital and the mental health of children in rural China with different experiences of parental migration. Soc Sci Med. (2015) 132:270–7. 10.1016/j.socscimed.2014.10.050 25465499

[50] Li C Zhang Q Li N. Does social capital benefit resilience for left-behind children? an evidence from mainland China children and youth services. Review. (2018) 93:255–62. 10.1016/j.childyouth.2018.06.033

[51] Putnam RD. The prosperous community:social capital and public life. Am Prospect. (1993) 3:13.

[52] Hu Y Han J Chen X Yang S Xu Y Xie S. Study on the Psychological-health Status and Its Relationship with Social Capital Among Left-behind Children in Rural Area Macheng, Hubei Province. Chinese J Epidemiol. (2014) 35:31–4. 24685033

[53] Cai H Wang S. The influence of urban community correction on neighborhood cohesion. Academic Monthly. (2021) 53:125–38. 10.19862/j.cnki.xsyk.000131

[54] Cai H Xu J. Urban Community and Resident Health: a Multi-layered Analysis Based on the 2018 CLDS Data. Shandong Soc Sci. (2022) 2:176–85. 10.14112/j.cnki.37-1053/c.2022.02.009

[55] Radloff LS. The CES-D Scale: a self-report depression scale for research in the general population. Appl Psychol Meas. (1977) 3:385–401. 10.1177/01466216770010030623302475

[56] Jiang L Wang Y Zhang Y Li R Wu H Li C. The reliability and validity of the center for epidemiologic studies depression scale (CES-D) for chinese university students. Front Psychiatr. (2019) 8:10. 10.3389/fpsyt.2019.00315 31178764 PMC6537885

[57] Blodgett JM Lachance CC Stubbs B Co M Wu Y Prina M. A systematic review of the latent structure of the Center for Epidemiologic Studies Depression Scale (CES-D) amongst adolescents. BMC Psychiatr. (2021) 21:1. 10.1186/s12888-021-03206-1 33874939 PMC8054366

[58] Dozeman E van Schaik DJF van Marwijk HWJ Stek ML Van Der Horst HE F AAT. The Center for Epidemiological Studies Depression Scale (CES-D) is an adequate screening instrument for depressive and anxiety disorders in a very old population living in residential homes. Int J Geriatr Psychiatr. (2011) 239-246. 10.1002/gps.2519 20623777

[59] Zhang Y Ting RZW Lam MHB Lam S Yeung RO Nan H. Measuring depression with CES-D in Chinese patients with type 2 diabetes: the validity and its comparison to PHQ-9. BMC Psychiatr. (2015) 15:580. 10.1186/s12888-015-0580-0 26281832 PMC4538746

[60] Cheng S Chan ACM. The center for epidemiologic studies depression Scale in older Chinese: thresholds for long and short forms. Int J Geriatr Psychiatry. (2005) 20:465–70. 10.1002/gps.1314 15852439

[61] Vilagut G Forero CG Barbaglia G Alonso J. Screening for depression in the general population with the center for epidemiologic studies depression (CES-D): a systematic review with meta-analysis. PLoS One. (2016) 11:e155431. 10.1371/journal.pone.0155431 27182821 PMC4868329

[62] Yang L Jia C Qin P. Reliability and validity of the Center for Epidemiologic Studies Depression Scale (CES-D) among suicide attempters and comparison residents in rural China. BMC Psychiatr. (2015) 15:1. 10.1186/s12888-015-0458-1 25886490 PMC4426543

[63] Chin WY Choi EPH Chan KTY Wong CKH. The psychometric properties of the center for epidemiologic studies depression scale in chinese primary care patients: factor structure, construct validity, reliability, sensitivity and responsiveness. PLoS One. (2015) 10:e135131. 10.1371/journal.pone.0135131 26252739 PMC4529142

[64] Zhang B Fokkema M Cuijpers P Li J Smits N Beekman A. Measurement invariance of the center for epidemiological studies depression scale (CES-D) among Chinese and Dutch elderly. BMC Med Res Methodol. (2011) 11:74. 10.1186/1471-2288-11-74 21595982 PMC3123656

[65] Jin J Shi Y Zhu L. The barriers of identity: Population diversity, social trust, and crime. Chinese J Sociol. (2020) 40:191–216. 10.1177/2057150X221091078

[66] Zhou S Cheung M. Hukou system effects on migrant children's education in China: learning from past disparities. Int Soc Work. (2017) 60:1327–42. 10.1177/0020872817725134

[67] Karlson KB Holm A Breen R. Comparing regression coefficients between same-sample nested models using logit and probit: a new method. Sociol Methodol. (2012) 12:286–313. 10.1177/0081175012444861

[68] Karlson KB Holm A. Decomposing primary and secondary effects: a new decomposition method. Res Soc Stratif Mobil. (2011) 29:221–37. 10.1016/j.rssm.2010.12.005

[69] Kohler U Karlson KB Holm A. Comparing coefficients of nested nonlinear probability models. Stata J. (2011) 11:420–38. 10.1177/1536867X1101100306

[70] Breen R Karlson KB Holm A. Total, Direct, and Indirect Effects in Logit and Probit Models. Sociologic Methods Res. (2013) 13:164. 10.1177/004912411349457230256434

[71] Xiao Y He L Chen Y Wang Y Chang W Yu Z. Depression and deliberate self-harm among Chinese left-behind adolescents: a dual role of resilience. Asian J Psychiatr. (2020) 48:101883. 10.1016/j.ajp.2019.101883 31786362

[72] Zhang H Chi P Long H Ren X. Bullying victimization and depression among left-behind children in rural China: roles of self-compassion and hope. Child Abuse Negl. (2019) 96:104072. 10.1016/j.chiabu.2019.104072 31319239

[73] Guo J Chen L Wang X Liu Y Chui CHK He H. The relationship between internet addiction and depression among migrant children and left-behind children in China. Cyberpsychol Behav Soc Network. (2012) 15:585–90. 10.1089/cyber.2012.0261 23002986

[74] Bowlby J. Attachment and loss: retrospect and prospect. Am J Orthopsychiatr. (1982) 52:664–78. 10.1111/j.1939-0025.1982.tb01456.x 7148988

[75] Carnelley KB Rowe AC. Repeated priming of attachment security influences later views of self and relationships. Pers Relatsh. (2007) 14:307–20. 10.1111/j.1475-6811.2007.00156.x

[76] Gillath O Shaver PR. Effects of attachment style and relationship context on selection among relational strategies. J Res Pers. (2007) 41:968–76. 10.1016/j.jrp.2006.11.003

[77] Ai H Hu J. Psychological resilience moderates the impact of social support on loneliness of “left-behind” children. J Health Psychol. (2016) 21:1066–73. 10.1177/1359105314544992 25139895

[78] Guang Y Feng Z Yang G Yang Y Wang L Dai Q. Depressive symptoms and negative life events: What psycho-social factors protect or harm left-behind children in China? BMC Psychiatr. (2017) 17:1554. 10.1186/s12888-017-1554-1 29246120 PMC5732424

[79] Puetz VB Kohn N Dahmen B Zvyagintsev M Schuppen A Schultz RT. Neural response to social rejection in children with early separation experiences. J Am Acad Child Adolesc Psychiatry. (2014) 53:1328–37. 10.1016/j.jaac.2014.09.004 25457931

[80] Su S Li X Lin D Zhu M. Future orientation, social support, and psychological adjustment among left-behind children in rural china: a longitudinal study. Front Psychol. (2017) 8:1309. 10.3389/fpsyg.2017.01309 28824493 PMC5539116

[81] Xing H Yu W Xu F Chen S. Influence of social support and rearing behavior on psychosocial health in left-behind children. Health Qual Life Outcomes. (2017) 15:592. 10.1186/s12955-017-0592-1 28103881 PMC5248492

[82] De Wolff MS van Ijzendoorn MH. Sensitivity and attachment: a meta-analysis on parental antecedents of infant attachment. Child Dev. (1997) 68:571–91. 10.1111/j.1467-8624.1997.tb04218.x 9306636

[83] Yue A Shi Y Luo R Wang B Weber A Medina A. Stimulation and early child development in China: caregiving at Arm's Length. J Develop Behav Pediatr. (2019) 40:458–67. 10.1097/DBP.0000000000000678 31107768

[84] Yue A Shi Y Luo R Chen J Garth J Zhang J. China's invisible crisis: cognitive delays among rural toddlers and the absence of modern parenting. China J (Canberra, ACT). (2017) 78:50–80. 10.1086/692290

[85] Luo R Jia F Yue A Zhang L Lyu Q Shi Y. Passive parenting and its association with early child development. Early Child Dev Care. (2017) 189:1709–23. 10.1080/03004430.2017.1407318

[86] Wang B Luo X Yue A Tang L Shi Y. Family environment in rural china and the link with early childhood development. Early Child Dev Care. (2022) 192:617–30. 10.1080/03004430.2020.1784890

[87] Wang L Liang W Zhang S Jonsson L Li M Yu C. Are infant/toddler developmental delays a problem across rural China? J Comparat Econ. (2019) 47:458–69. 10.1016/j.jce.2019.02.00330360569

[88] Zhao X Sun H Zhang X Deng X. Analysis of China's youth health policy and work progress since the implementation of the medium and long-term youth development plan. China Youth Study. (2020) 20:38–47. 10.19633/j.cnki.11-2579/d.2020.0177

